# Years of healthy life lost due to adverse pregnancy and childbirth outcomes among adolescent mothers in Thailand

**DOI:** 10.3934/publichealth.2018.4.463

**Published:** 2018-12-07

**Authors:** Ei Ei Aung, Tippawan Liabsuetrakul, Warisa Panichkriangkrai, Nuttapat Makka, Kanitta Bundhamchareon

**Affiliations:** 1 International Health Policy Program, Ministry of Public Health, Nonthaburi, THAILAND; 2 Epidemiology Unit, Faculty of Medicine, Prince of Songkla University, Hat Yai, Songkhla, THAILAND

**Keywords:** disability adjusted life years, Year of Life Lost, Year Lived with Disability, maternal, adolescent, pregnancy and childbirth, Thailand

## Abstract

**Background:**

Preventing adolescent pregnancy and childbirth is one of the targets of Sustainable Development Goals. Measuring the burden pregnancy and childbirth places on adolescents is required to convince society and decision makers that this is an important goal.

**Objective:**

This study aimed to estimate (1) the years of healthy life lost due to adolescent pregnancy and childbirth in terms of disability adjusted life years (DALYs), (2) the contribution of adolescent pregnancy and childbirth to the total DALYs lost from all reproductive ages, and (3) the magnitude of the burden due to five main direct obstetric causes and sequelae in adolescent mothers in Thailand in 2014.

**Methods:**

Data were retrieved from a national in-patient registered database and a vital registration database. Health consequences of five main direct obstetric causes were extracted from the Global Burden of Diseases (GBD) 2000 study. The DALYs were calculated by the combination of Years of Life Lost (YLL) due to premature death and Years Lived with Disability (YLD) due to adverse pregnancy and childbirth in adolescent mothers.

**Results:**

There were a total of 2599 years of DALYs lost from the consequences of adolescent pregnancy and childbirth, and unsafe abortion resulted in the highest burden. Mortality was the primary driver for the total DALYs lost with 1704 years, and maternal hemorrhage dominated in the total YLL. Unsafe abortion contributed the highest burden to nonfatal morbidity. Obstructed labor commonly occurred in adolescent pregnancies.

**Conclusion:**

Among the DALYs lost due to pregnancy and childbirth for all reproductive aged women, 15.4% were attributed to adolescents. The five main obstetric causes of mortality and morbidity are all preventable conditions. Increased efforts from all stakeholders are essential to implement appropriate interventions to minimize adverse health outcomes in adolescent mothers.

## 1. Introduction

Globally, in 2008, 16 million births occurred in adolescents aged 15–19 years, among which more than 90% occurred in low- and middle-income countries (LMICs) [1]. The adolescent period is the transition period from childhood to adulthood. The World Health Organization (WHO) defines adolescence as the period from 10–19 years of age, and it is divided into an early adolescent period (ages 10–14 years) and a late adolescent period (ages 15–19 years). Adolescence is the period of biological growth with the onset of puberty inducing both sexual and reproductive maturation, resulting in physical, psychological and social changes as well as the possibility of pregnancy and childbirth [2],[3]. The consequences of adolescent pregnancy and childbirth are serious because of the health and societal burden caused by long-term health care costs for the mothers and their children and their high rate of school dropout [4],[5],[6]. Both an adolescent and her baby have a higher risk of morbidity and mortality than a baby born to a mother aged 20–24 years [1].

In Thailand, pregnancy-related hospital admission is the leading cause of all hospital admissions among females, and 35.9% of all female admissions were of adolescents in 2010 [7]. According to the United Nations Population Fund 2013 report, approximately 1.1 million adolescents aged 15 to 19 years are married and/or sexually active, and Thailand's adolescent pregnancy rate is the fifth-highest in the Association of Southeast Asian Nations (ASEAN) [8]. Although the adolescent birth rate in Thailand is lower than the average rate among the countries in the Southeast Asia Region at 54 births per 1000 adolescents aged 15–19 years, this rate has increased from 40.7 in 1992 to 47.9 in 2014. The adolescent birth rate was highest among adolescents aged 18–19 years, probably because these ages are in the legal range of marriage [9]. Thailand's National Health Plan has targeted a reduction in the prevalence of teenage pregnancies to less than 10% of all pregnancies [10]. Although the success of the family planning programs and services in Thailand has been acknowledged worldwide, and the prevalence of contraceptive use among all Thai reproductive age women had increased to 79.3% in 2012 [11], the proportion of adolescent births was still higher than the national target. Moreover, according to the 2012 Multiple indicator cluster survey data, 14.7% of Thai women aged 20–49 reported that they were married before aged 18 years [12] and 11.7% of adolescent mothers aged 15–19 years had given birth more than once [8]. Additionally, adolescent mothers were less likely to attend their first prenatal visit during their first trimester compared to adult mothers (16% vs 38.9%) [5]. As a result, targeting pregnancy and childbirth in adolescence needs to be prioritized, and the magnitude of the burden of pregnancy and childbirth in adolescence needs to be revealed.

Adverse pregnancy and childbirth outcomes due to direct or indirect obstetric complications have been reported in previous studies; however, very few studies have measured the burden of fatal and nonfatal health outcomes of pregnancy and childbirth in terms of Disability Adjusted Life Years (DALYs) [5],[13],[14],[15],[16],[17],[18]. The analysis of DALYs lost can show how many healthy years of life are lost: the number of years lost due to not only premature death but also the burden of morbidities due to ill-health or disease conditions [19]. Moreover, the use of DALYs provides evidence of burdens with implications in the context of population health, priority setting and intervention evaluations [20]. According to a previous global burden of diseases study in 2002, the healthy lives lost due to maternal conditions contributed 0.4% and 16.3% to total DALYs lost among those aged 5–14 and 15–29 years, respectively [17]. The burden of adolescent pregnancy accounted for 23% of total DALYs lost due to pregnancy and childbirth in all age groups [18].

This study thus aimed to estimate (1) the loss of healthy years of life due to adolescent pregnancy and childbirth in Thailand during 2014 in terms of DALYs, (2) its contribution to the total burden of disease among females of reproductive age, and (3) the magnitude of the burden due to five main direct obstetric causes and their sequelae.

## 2. Methods

### 2.1. Study design and data sources

This secondary data analysis as a part of the Thailand Burden of Diseases and Injuries Study 2014 was conducted using the databases from the National Civil Registration (Vital Registration system) and an in-patient registered database in 2014. Adolescents aged 10–19 years who were pregnant or had delivered were included and their information retrieved from the databases. We classified the adolescents into two age groups: 10–14 years of age and 15–19 years of age.

The cases and consequences of pregnancy and childbirth related mortality and morbidity were recorded based on the Tenth Revision of the International Classification of Diseases (ICD-10) codes [21] shown in Table 1.

**Table 1.** ICD-10 codes for maternal conditions to estimate the burden of adolescent pregnancy and childbirth.

Maternal Conditions	ICD-10 code
Maternal hemorrhage	O44-O46, 067, 072
Maternal sepsis	O85-O86
Hypertension in pregnancy	O10-O16
Obstructed labor	O64-O66
Abortion	O00-O08
Other maternal conditions	O20-O43, O47-O63, O68-O71, O73-O75, O87-O99

**Figure 1.**
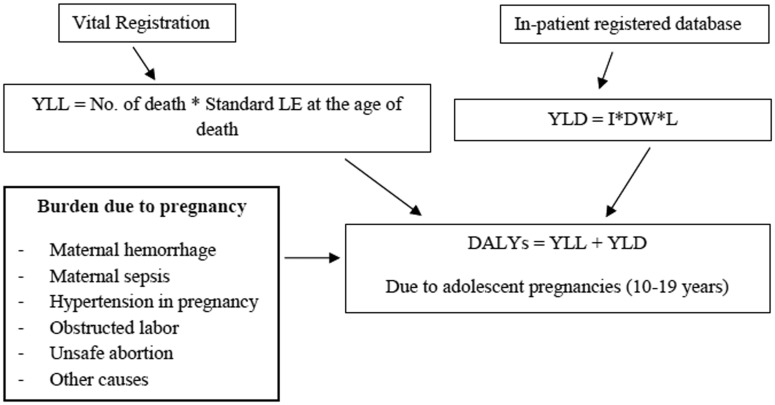
Conceptual framework for analyzing the burden of adolescent pregnancy and childbirth. YLL: Year of Life Loss (due to premature mortality), LE: Life Expectancy, YLD: Year Lived with Disability, I: Incidence, DW: Disability Weight, L: Average length of disability till remission or death (in year), DALY: Disability Adjusted Life Years.

Maternal death refers to the death of a woman while pregnant or within 42 days of termination of pregnancy, irrespective of the duration and site of the pregnancy, from any cause related to or aggravated by the pregnancy or its management, but not from accidental or incidental causes, according to the WHO definition [22]. The National Civil Registration 2014 was chosen to be the main source of mortality statistics. A self-reported interview survey, the intercensal survey (Survey of Population Change, 2005–2006) was used to assess the completeness of the death registration. Accuracy of the cause of death from vital registration was ensured by using the 2005 nation-wide verbal autopsy study from the Setting Priority using Information on Cost-Effectiveness (the SPICE study) that has been detailed elsewhere [16]. The National In-patient Registered Database of 2014 was utilized as an important source of data for estimating the number of cases and morbidity burden of five major cases and their consequences, namely, maternal hemorrhage, maternal sepsis, hypertension in pregnancy (pregnancy induced hypertension), obstructed labor, unsafe abortion, and other maternal complications as shown in Figure 1. The morbidity burden for other maternal conditions were estimated based on mortality due to other maternal conditions caused by adolescent pregnancy and childbirth. To estimate the burden, data from adolescents who visited a hospital for pregnancy and childbirth were adjusted with the health facility-based dataset in order to cover all types of hospitals. Local data sources were extensively reviewed based on the suggestions made by external experts to derive the best estimate of causes and sequelae. The data justifications were selected after critical examination of the incidence and prevalence of maternal disorder consequences of neighboring countries and nearby regions.

### 2.2. Data analysis

The burden of adolescent pregnancy and childbirth in this analysis was measured by Disability Adjusted Life Years (DALYs), which is calculated by the combination of Years of Life Lost (YLL) due to premature death and Years Lived with Disability due to specific nonfatal conditions (YLD). The calculating formula is DALYs = YLL + YLD. One DALY means one year of healthy life lost. The DALY is described in detail in Murray and Lopez (1996) [23]. YLL is determined by the average life expectancy at the age of death. We used a standard life expectancy derived from the Coale and Demeny West Model 26 life table. The simplified formula of YLL is YLL = Σdx*ex, where ex is the expected life at the age x based on standard life expectancy and dx is the number of deaths at the age x (number of deaths by causes).

The basic formula for calculating YLD is YLD = I*DW*L, where I is the number of incident cases in the reference period, DW is the disability weight and L is the average length of disability until remission or death measured in years. Therefore, it is necessary to determine the incidence of obstetric complications and their consequences.

Disability weight is an important part of the DALY calculation and indicates the valuation of the health state. The scale of DW is from zero (perfect health) to one (worst possible health state). Disability weights for all causes and consequences of maternal conditions were obtained from the GBD 2000 study [19] and an Australian study [24].

The disease sequelae and duration were considered according to the GBD 2000. The disease sequelae, disability weight and duration used in the present study are listed in Table 2, and we estimated the proportion of sequelae cases for each major cause from the obstetrics and gynecology experts' suggestions and literature reviews.

**Table 2.** Causes and consequences of adolescent pregnancy and childbirth, disability weight and duration for YLD estimation of five major direct causes and sequelae.

	Causes and Sequelae	Disability weight (from the GBD study [19] and an Australian study [24])	Duration (year)
1.	Maternal Hemorrhage		
	Case episode	0.011 (GBD) for moderate anemia	3 months (0.25)
	Severe anemia after postpartum hemorrhage	0.093 (GBD)	1 month (0.08)
	Caesarean section delivery (due to antepartum hemorrhage)	0.349 (Australian study)	2 weeks (0.04)
2.	Maternal Sepsis		
	Case episodes	0.500 (GBD)	2 weeks (0.04)
	Infertility due to maternal sepsis	0.180 (GBD)	Remaining reproductive life
3.	Hypertension in Pregnancy		
	Case episode	0.117 (Australian study)	2 months (0.17)
	Neurological sequelae following pre-eclampsia	0.388 (GBD)	Remaining life expectancy
	Caesarean section delivery (due to pregnancy induced hypertension)	0.349 (Australian study)	2 weeks (0.04)
4.	Obstructed labor		
	Caesarean section delivery (due to obstructed labor)	0.349 (Australian study)	2 weeks (0.04)
5.	Unsafe abortion		
	Pelvic inflammatory disease (due to unsafe abortion)	0.5 (GBD)	2 weeks (0.04)
	Infertility who still have child wish	0.18 (GBD)	Remaining reproductive life

## 3. Results

A total of 711,805 live births were registered in the birth registration of Thailand in 2014. There were 115,490 live births to adolescents, including 3,213 live births to adolescents aged 10–14 years and 112,277 to those aged 15–19 years, and these accounted for 16.2% of all live births. The adolescent live birth rate was 1.6 and 47.9 per 1000 adolescents aged 10–14 and 15–19 years, respectively.

### 3.1. Healthy life lost due to adolescent pregnancy and childbirth

Total YLL, YLD, and DALYs due to pregnancy and childbirth complications among the adolescents aged 10–19 years are shown in Table 3. There were a total of 2,599 years of healthy life lost due to the consequences of adolescent pregnancy and childbirth in 2014, and these mainly affected mothers aged 15–19 years. Five main direct causes and their consequences contributed to approximately 78% of the total burden. Among the five main causes of pregnancy and childbirth, unsafe abortion resulted in the highest DALYs lost in both adolescents aged 10–14 years and 15–19 years with a total of 725 years lost. Maternal hemorrhage caused the second highest level of burden followed by maternal sepsis in the total DALYs lost for adolescent pregnancies among those aged 15–19 years, but maternal sepsis caused the second highest burden in the 10–14 years age group. The burden of the two main causes: unsafe abortion and maternal hemorrhage with consequences accounted for more than half of the total burden from adolescent pregnancy and childbirth.

Regarding the fatal and nonfatal burden of adolescent pregnancy and childbirth, mortality was the primary driver of years of healthy life lost and accounted for 65.6% of the total burden. According to the current analysis, there was no death reported among adolescents aged 10–14 years. However, 26 deaths occurred in adolescents aged 15–19 years, leading to an adolescent-specific maternal mortality ratio of 23 per 100,000 live births with all of the YLL counts of 1704 years. Five main direct causes contributed to approximately 73% of total healthy life lost due to premature death. Maternal hemorrhage was the leading cause of death with 590 years of healthy life lost, followed by maternal sepsis and unsafe abortion (262 years lost due to each). There was no death due to obstructed labor in both adolescent groups. The nonfatal morbidity burden due to adolescent pregnancy and childbirth accounted for 856 years and 34.4% of the total DALYs lost for adolescents aged 10–19 years old. Unsafe abortion resulted in the largest burden, resulting in more than half of the morbidity burden of pregnancy and childbirth in both adolescent age groups. Different from the mortality burden, a higher nonfatal morbidity burden was caused by maternal sepsis and hypertension in pregnancy than by maternal hemorrhage.

**Table 3.** Total YLL, YLD, and DALYs due to pregnancy and childbirth complications in adolescents aged 10–19 years.

Causes with sequelae	No. of death		Death rate per 100,000 live births (LBs) of 15–19 years (LBs - 112277)	Total YLL (years)			Total numbers of case		Rate per 100,000 live births		Total YLD			Total DALYs		
	10–14 years	15–19 years		10–14 years	15–19 years	10–19 years	10–14 years	15–19 years	10–14 years	15–19 years	10-14 years	15–19 years	10–19 years	10–14 years	15–19 years	10–19 years (%)*
Maternal hemorrhage	0	9	8	0	590	590	114	3647	3546	3248	1	17	18	1	607	608 (23.4)
Maternal sepsis	0	4	3	0	262	262	41	840	1272	749	6	109	115	6	371	377 (14.5)
Hypertension in pregnancy	0	2	2	0	131	131	146	4317	4552	3845	4	104	108	4	235	239 (9.2)
Obstructed labor	0	0	0	0	0	0	174	5472	5427	4874	2	73	75	2	73	75 (2.9)
Unsafe abortion	0	4	3	0	262	262	35	675	1089	602	26	437	463	26	699	725 (27.9)
Other maternal conditions	0	7	6	0	459	459					0	116	116	0	575	575 (22.1)
Total (%)*	0	26	23	0	1704	1704 (65.6)					39	856	895 (34.4)	39	2560	2599 (100)

Note: YLL: Year Life Loss, YLD: Year Lived with Disabilities, DALYs: Disability Adjusted Life Years. (%) *: Percentage in total DALYs lost among those aged 10–19 years. YLD: counts of other maternal conditions were obtained from a burden of diseases and injuries study Thailand, 2014. NB: All numbers are rounded to absolute values.

**Table 4.** Attribution of YLL, YLD, DALYs lost from adolescent pregnancy and childbirth in the total burden of obstetric outcomes by all reproductive ages.

Five major direct causes and other conditions	YLL			YLD			DALYs		
	All reproductive ages	10–19 years	% of 10–19 years in all reproductive ages	All reproductive ages	10–19 years	% of 10–19 years in all reproductive aged	All reproductive ages	10–19 years	% of 10–19 years in all reproductive aged
Maternal hemorrhage	2439	590	24.2	173	17	9.9	2611	607	23.2
Maternal sepsis	1075	262	24.4	337	115	34.2	1412	378	26.7
Hypertension in pregnancy	954	131	13.7	952	108	11.3	1906	239	12.5
Obstructed labor	0	0	0	434	75	17.5	434	76	17.5
Unsafe abortion	1663	262	15.8	1779	463	26.0	3442	725	21.1
Other maternal conditions	6113	459	7.5	958	116	12.1	7070	575	8.1
Total	12243	1704	13.9	4632	895	19.3	16876	2599	15.4

Note: YLL: Year Life Loss, YLD: Year Lived with Disabilities, DALYs: Disability Adjusted Life Years. NB: All numbers are rounded to absolute values.

### 3.2. Burden on adolescents in the overall DALYs of obstetric complications

The proportion of the burden on adolescents to the total burden of pregnancy and childbirth from all reproductive ages is presented in Table 4. According to the burden of diseases study 2014, the total burden of pregnancy and childbirth outcomes from all reproductive age women was 16876 years and 15.4% of this number was contributed by adolescents. About one-fourth of the burden of maternal hemorrhage and maternal sepsis in all reproductive women was contributed by adolescents. Within the total burden of premature death from maternal hemorrhage and sepsis, almost 25% were contributed by adolescent pregnancy and childbirth. Almost 20% of the total morbidity burden from pregnancy and childbirth was placed on adolescents, and maternal sepsis was the most dominant in overall morbidity burden.

### 3.3. Magnitude of the five main direct obstetric causes and their sequelae

Obstructed labor was the most common complication in both adolescents aged 10–14 years and 15–19 years, followed by hypertension during pregnancy and maternal hemorrhage among the five major direct pregnancy-related complications (Table 3). Higher rates of all five main direct causes per 100,000 live births were seen in adolescents aged 10–14 years compared with those aged 15–19 years.

## 4. Discussion

The burden experienced by Thai adolescents during pregnancy and childbirth accounts for 15.4% of the pregnancy and childbirth related burden of all reproductive aged women. The burden of maternal disorders was in total DALYs 60 per 100,000 of the female population aged 10–19 years. Unsafe abortion, maternal hemorrhage and maternal sepsis demonstrated noticeable burdens with YLL, YLD and DALYs measures in both adolescent age groups.

Unsafe abortion dominated the total DALYs lost to adolescent pregnancy and the childbirth burden, followed by maternal hemorrhage in Thailand. The reason for this is that abortion is illegal in Thailand except in cases of risk to a woman's health or if the pregnancy is the result of rape or other sexual crimes, but those who seek abortions due to other reasons will find abortions through whatever means they can [25]. Abortions with complications in adolescents might be due to lack of proper reproductive health knowledge, peer pressure, physiologic immaturity, low socioeconomic status and risk taking behaviors [8],[26]. The results from this study are similar to the findings from the Korean burden of diseases estimation of maternal disorders, where unsafe abortion and maternal hemorrhage dominated the DALYs loss among maternal disorders in the adolescent group [27]. The GBD study on child and adolescent health also reported that abortion, ectopic pregnancy, and/or miscarriage were the most common disabling outcomes of adolescent pregnancy [28]. According to the WHO Adolescent Pregnancy Factsheet 2008, 14% of total unsafe abortion cases in LMICs were attributed to adolescents aged 15–19 years [18]. Approximately 2.5 million adolescents undergo unsafe abortions every year, and adolescents are seriously affected by its complications, more than older women [18]. Worldwide, the burdens due to unsafe abortion and maternal sepsis rank fourth and sixth, respectively, among the total DALYs of all burdens of diseases in females aged 15–19 years [29].

The GBD study from the Institute for Health Metrics and Evaluation reported that adolescent mothers under 20 years of age contributed 8.7% of total DALYs lost due to the burden of maternal disorders in Thailand, 2014 [30]. However, the total DALYs lost reported in that study differs from our study. The GBD study used a prevalence-based approach, while our study applied an incidence-based approach. Hence, the DALYs count must be affected by these different approaches [31], the data sources used for burden estimations [32] and the considered sequelae of each maternal cause. The study conducted by Wagner et al. (2015) reported that the YLD result from the prevalence-based approach was 29–38% less than incidence-based approach estimation [33].

In our study, the maternal mortality ratio among adolescents aged 15–19 years was 23 per 100,000 live births. This finding was higher than the results of another study [12] that was conducted in the same year 2014 in Thailand (16.9 per 100,000 live births). This could be explained by the evaluation of the maternal deaths in the two studies being different in approach, where the other study used multiple administrative data sources [12]. However, the findings of the current study showed a lower mortality rate than the Southeast Asia regional rate of 130 (CI: 48–210) per 100,000 live births in the adolescent group [34] where the study performed an estimation of the age-aggregated mortality rate based on the proportion of maternal deaths among deaths of reproductive aged women. Depending on the data sources and approaches used for analysis, there can be differences in the maternal mortality ratio [35].

Obstetric complications were the leading causes of deaths in adolescents aged 15–19 years, which was in accordance with the report of WHO and a review study in LMICs (2016) [36],[37]. The most common maternal-related causes of death were hypertensive disorders, hemorrhage, unsafe abortion and sepsis, which are similar to the findings of the Neal et al. study (2016) [37]. In Southeast Asia, 30% of all maternal deaths for all reproductive aged women were due to hemorrhage [38]. Additionally, the GBD study reported that maternal hemorrhage was the highest-ranked cause of maternal mortality among adolescents aged 10 to 19 years, especially in low sociodemographic index geographical areas [28].

Similar to a previous study [39], this study also demonstrated obstructed labor as the most common complication during delivery in adolescents aged 10–14 years when compared to other age groups. In one multicountry study by Ganchimeg et al. (2013), the authors also found that there was an increased risk of caesarean section due to obstructed labor with cephalopelvic disproportion in girls aged ≤15 years old [4], and this is due to the under-developed pelvis in young girls. Although the rate of obstructed labor per 100,000 live births was the highest in adolescents aged 10–14 and 15–19 years, its burdens associated with YLL, YLD and DALYs were not prominent. Increasing availability and accessibility of emergency obstetric services are strongly associated with the outcomes of obstructed labor [40]. In Thailand, the utilization of sexual and reproductive health services has clearly improved under the expansion of primary health care and universal health coverage [41]. That is a reason why the years of healthy life lost due to premature death from obstructed labor was lower even those these cases were common in adolescent mothers.

Although indirect obstetric causes are not included in this analysis, the burden from these causes should not be neglected. From a nationwide survey, the prevalence of postpartum depression was 8.4% among all mothers who delivered within six to eight weeks (6.8% for those aged <20 years) [42]. Additionally, according to the Thailand Burden of Diseases and Injuries Study in 2014, 21.7% of all DALYs lost from low birth weight infants were attributed to infants born from adolescent mothers [43]. Those negative health outcomes are also important and need to be considered in future burden estimations and can be reduced by mothers receiving appropriate perinatal care [44].

The health burden from maternal-related causes may differ across regions and countries. The burden of adolescent pregnancy and childbirth (in a Thai population) in this study was estimated from the most reliable and available health report of mortality and morbidity under the public health insurance system at the national level. The findings of this study confirmed that appropriate detection and management of the most common causes such as unsafe abortion, maternal hemorrhage, and sepsis are important for reducing the healthy lives lost from adolescent pregnancy and childbirth.

The prevalence of adolescent pregnancies in the current study is higher than the national aim and it indicates a failure or inefficiency of the reproductive health services. In addition, the higher rate of all five main direct complications per 100,000 live births in early adolescence than that in late adolescence should be an alert that the sexual and reproductive health programs directed at very young adolescents should be fairly distributed in comparison with those focused on older adolescents. The community-wide initiatives for providing sexual and reproductive health education and expanding access to a wider range of contraceptive methods that respond to adolescent needs is important to reduce the unwanted pregnancy and birth rate among adolescents [45]. Moreover, since abortion is illegal in Thailand, the burden from unsafe abortion is high compared to other causes. By relaxing abortion laws and allowing services to be provided more openly by skilled practitioners, we can reduce the rate of unsafe abortions. The roles of research, health providers, grassroots organizations, and media are essential to highlight the importance of liberalizing abortion laws. Furthermore, care givers need to become better trained in safer abortion methods and be able to transfer patients to appropriate healthcare centers when a complication arises. Therefore, essential reproductive health education should reach all young girls to prevent risky sexual behaviors, young marriages and unplanned pregnancy.

## 5. Limitations of the study

There were some limitations to our study. First, we used the routine diagnosis coding from the in-patient database and birth registration, which might be affected by a lack of coding consistency across the country. However, each of the public health insurance schemes audits both the out-patient and in-patient data of every hospital, and they found some errors in diagnostics and coding [46]. Second, although the verbal autopsy data were used for maternal death accuracy, the estimation might have some uncertainty since a large proportion of ill-defined causes of death are still challenged in Thailand [47]. Third, the estimated total burden of adolescent pregnancy and childbirth in this study did not include other burdens such as gender-based violence and maternal depression [42] since the authors followed the methods used in the GBD 2000 study. Therefore, the overall presented burden in this particular study might be underestimated.

## 6. Conclusions

Years of healthy life lost from adolescent mothers contributed to 15.4% of total DALYs lost from adverse pregnancy and childbirth outcomes among all reproductive aged women. Unsafe abortion and maternal hemorrhage were the most common causes of the burden of healthy life lost among all pregnancy and childbirth complications. Mortality was primarily driven by overall healthy lives lost, and maternal hemorrhage was the leading cause of death in adolescent pregnancies. The five main obstetric causes of mortality and morbidity are preventable conditions. Increased efforts from all stakeholders are essential to implement appropriate interventions to minimize adverse health outcomes in adolescent mothers.
